# Supplementary material for: The Preparation and Wear Behaviors of Phenol–Formaldehyde Resin/BN Composite Coatings
Source: Polymers (Basel). 2022 Oct 9;14(19):4230. doi: 10.3390/polym14194230 (PMC9572075; doi:10.3390/polym14194230)
Supplement: Supplementary file 1 [file polymers-14-04230-s001.zip › polymers-1931832-supplementary.pdf]

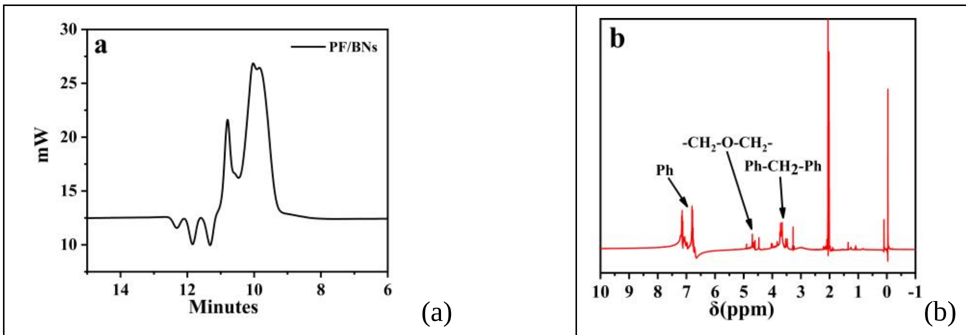

Figure S1. The GPC (a) and H-NMR (b) curves of phenolic

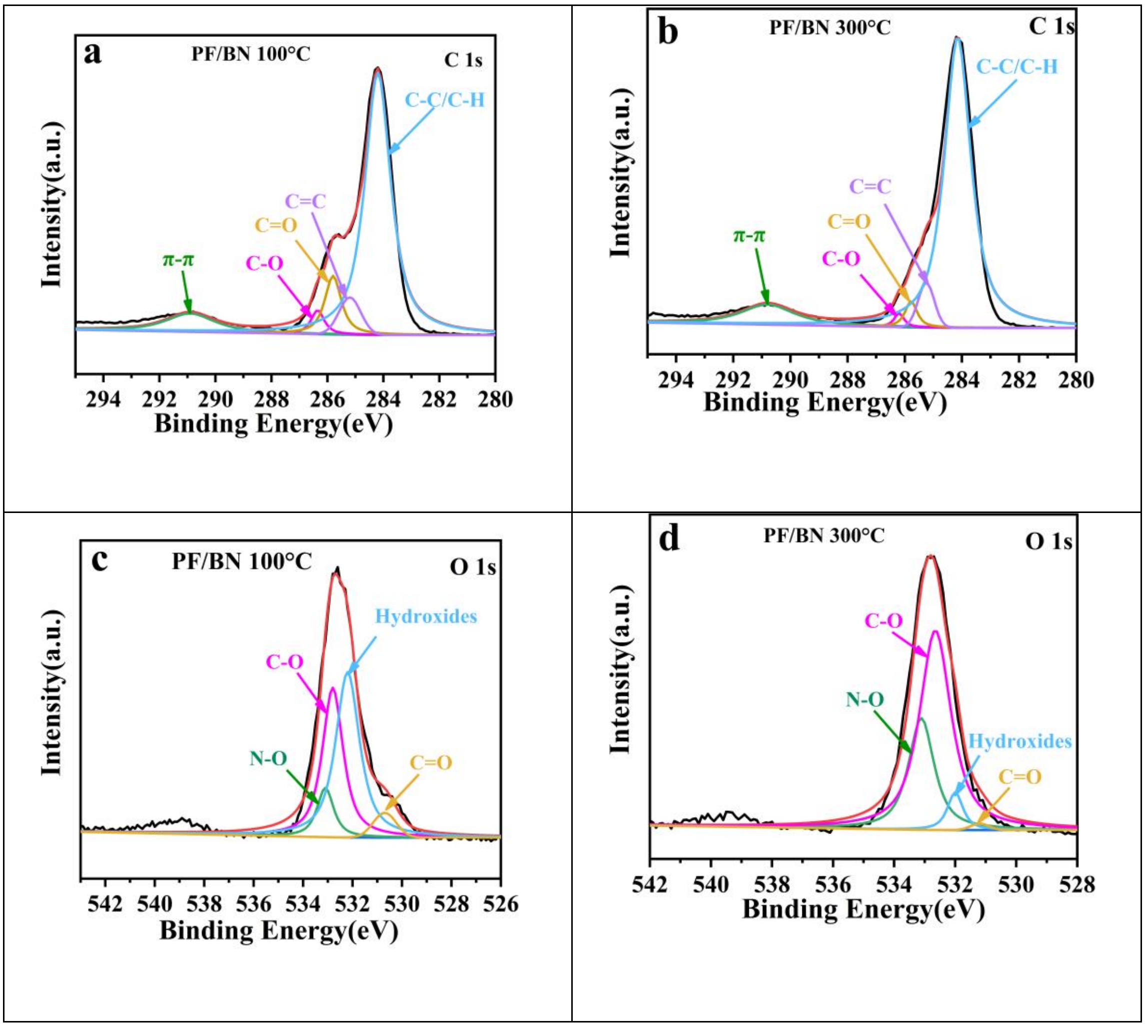

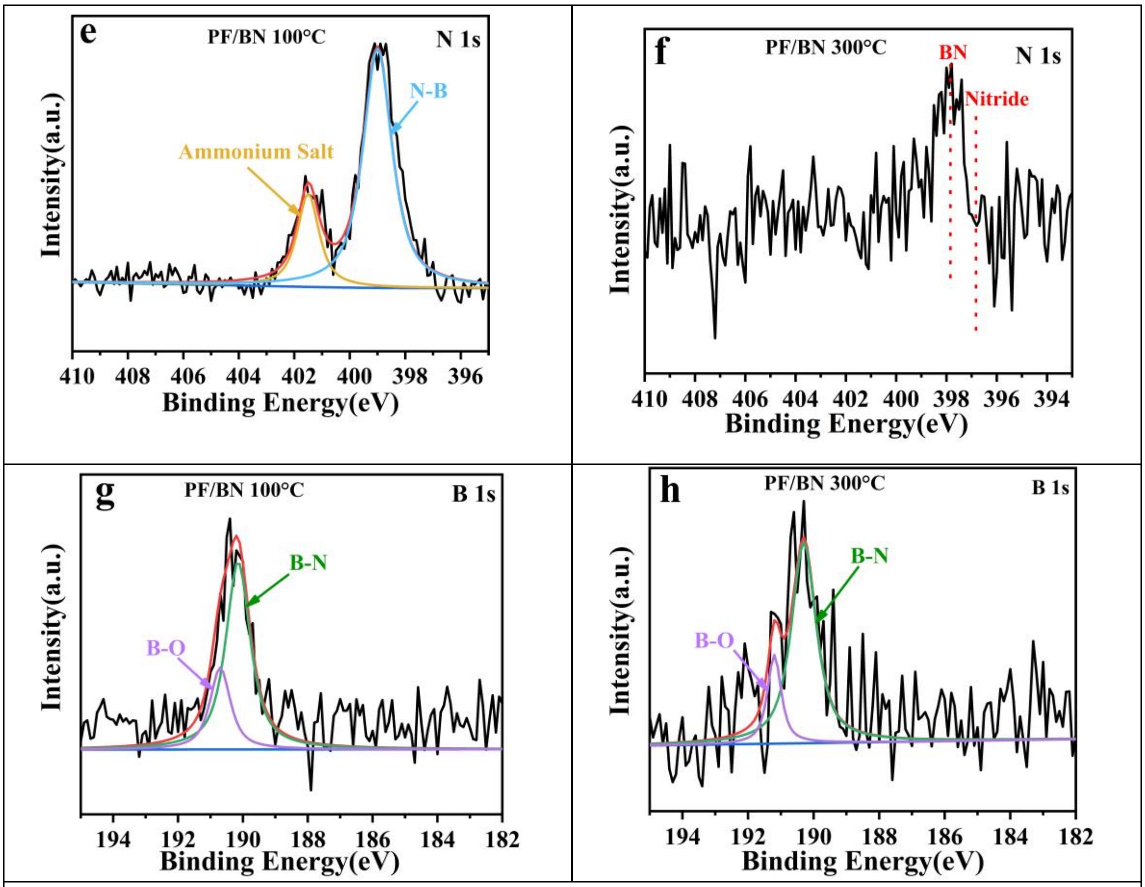

Figure S2. The XPS analysis of thePF/BN powders with heat treatment at 100 °Cand 300 °C, respectively

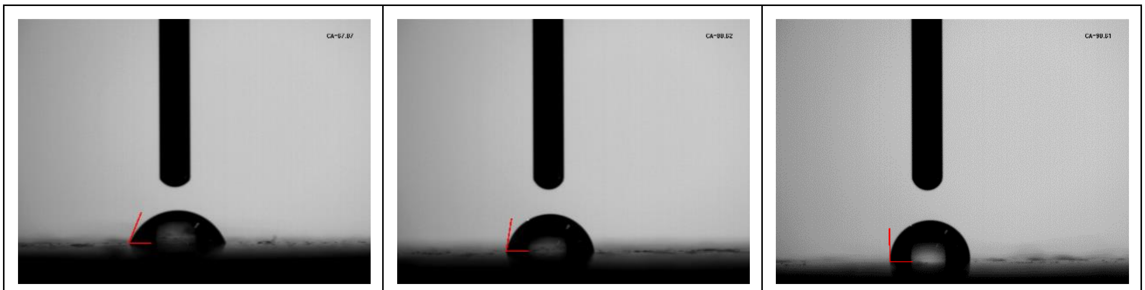

(a) (b) (c)

Figure S3 The contact angles of water drops on PF/BN films without (a) with heat treatment at 100 ℃ (b) and 300 ℃ (c), respectively

Formaldehyde	Formaldehyde is usually a colorless gas with a pungent smell. Easily soluble in water and diethyl ether, the concentration of aqueous solution up to 55%, and ethanol, acetone and other organic solvents according to any proportion of miscible, insoluble in petroleum ether. Liquid is easy to be turbid when stored for a long time in the cold, and the formation of triformaldehyde precipitation at low temperature. And formaldehyde also has reducing property
phenol	It can absorb moisture in the air and liquefy. It has a special smell, and the very dilute solution has a sweet taste. It is highly corrosive. Chemically reactive, solid at normal temperatures. Phenol is a phenolic substance with weak acidity and can react with alkali. Slightly soluble in cold water, mixed soluble in ethanol, ether, chloroform, glycerol
Hexagonal boron nitride (h-BN)	Hexagonal boron nitride (h-BN), is a white powder material, its crystal structure is very similar to graphite, and the physical and chemical properties of the two are also similar, so hexagonal boron nitride is also known as "white graphite", has important applications in heat conduction, lubrication, hydrogen storage and

	catalysis. It not only has the advantages of low density, high melting point, low hardness, anti-friction and good mechanical processing performance, but also has high temperature resistance, small coefficient of thermal expansion, high thermal conductivity, low dielectric constant, reliable electrical insulation and other excellent properties.

Table S1. The main physical and chemical properties of the raw materials
